# Overexpression of *CmMYB15* provides chrysanthemum resistance to aphids by regulating the biosynthesis of lignin

**DOI:** 10.1038/s41438-019-0166-y

**Published:** 2019-07-11

**Authors:** Cong An, Liping Sheng, Xinping Du, Yinjie Wang, Yi Zhang, Aiping Song, Jiafu Jiang, Zhiyong Guan, Weimin Fang, Fadi Chen, Sumei Chen

**Affiliations:** State Key Laboratory of Crop Genetics and Germplasm Enhancement, the Key Laboratory of Landscaping, Ministry of Agriculture, College of Horticulture, Nanjing Agricultural University, Nanjing, 210095 China

**Keywords:** Biotic, Transgenic plants, Transcriptional regulatory elements

## Abstract

MYB transcription factors are widely involved in the development of and physiological processes in plants. Here, we isolated the chrysanthemum R2R3-MYB family transcription factor *CmMYB15*, a homologous gene of *AtMYB15*. It was demonstrated that *CmMYB15* expression was induced by aphids and that CmMYB15 could bind to AC elements, which usually exist in the promoter of lignin biosynthesis genes. Overexpression of *CmMYB15* in chrysanthemum enhanced the resistance of aphids. Additionally, the content of lignin and the expression of several lignin biosynthesis genes increased. In summary, the results indicate that *CmMYB15* regulates lignin biosynthesis genes that enhance the resistance of chrysanthemum to aphids.

## Introduction

Members of the MYB family, one of the largest transcription factor families in plants, have a common conserved N-terminal MYB domain consisting of imperfect amino acid repeats of ~52 amino acids^1^. These members are generally divided into four classes, 1R-, R2R3-, 3R- and 4R-MYB proteins, according to the number of adjacent repeats, with the R2R3-MYB subfamily comprising one of the largest families of transcription factors in plants^2^. MYBs have been shown to be key factors involved in vital regulatory networks that regulate physiological and biochemical processes, for instance, plant development, responses to biotic and abiotic stress^3^, and primary and secondary metabolism, such as flavonoid biosynthesis and phenylpropanoid metabolism^4,5^.

The lignin biosynthetic pathway is among the important branches of the phenylpropanoid metabolic pathway, as lignin is the second most abundant plant biopolymer. Lignin is a major secondary cell wall component that provides mechanical support for plants and is also a chemical and physical barrier that provides defense against biotic and abiotic stress^6^. The precursors of lignin, which are phenolic compounds, can participate in plant-pathogen interaction pathways, such as that of the interaction between grapevine and *Erysiphe necator*^7^. A relatively high accumulation of lignin increases the strength of birch leaves, which reduces vulnerability to the autumnal moth, *Epirrita autumnata*^8^. Lignin enhances resistance to cell wall-degrading enzymes (CWDEs), which are produced by fungal necrotrophs, therefore preventing the diffusion of pathogen-produced toxins^9^. Enzymes participating in lignin biosynthesis include phenylalanine ammonia lyase (PAL), 4-hydroxycinnamoyl-CoA ligase (4CL), caffeoyl shikimate esterase (CSE), *p*-coumarate 3-hydroxylase (C3H), caffeoyl-CoA O-methyltransferase (CCoAOMT), cinnamoyl-CoA reductase (CCR), ferulate 5-hydroxylase (F5H), caffeic acid/5-hydroxyferulic acid O-methyltransferase (COMT), and cinnamyl alcohol dehydrogenase (CAD)^10,11^. Analysis of promoter sequences showed that most of these genes contain one or more AC-rich elements in their promoters, except for F5H^12^. Transcription factors such as MYB proteins regulate the expression of these genes by binding to AC elements. *AtMYB46* activates specific pathways of secondary cell wall biosynthesis and simultaneously upregulates *AtMYB85*, a downstream secondary cell wall-associated transcription factor^13^. *AtMYB58* and *AtMYB63* specifically activate lignin biosynthetic genes, which leads to the ectopic deposition of lignin in cells that are normally unlignified^6^. Similarly, *PtoMYB216*, an MYB-encoding gene in *Populus tomentosa*, was also found to activate lignin upstream biosynthetic genes^14^. On the other hand, *Eriobotrya japonica EjMYB1*, a transcriptional activator, and *EjMYB2*, a repressor, have an antagonistic relationship in terms of lignification, as the activator and repressor compete with each other for interaction with AC elements^15^. *PdMYB221* negatively regulates secondary cell wall formation by binding to the AC elements in the promoter of *PdCESA8*, *PdGT47C*, and *PdCOMT2*, leading to decreased lignin production^16^. Previous studies have indicated that several members of the MYB family regulate the synthesis of lignin. However, if other members of the MYB family are involved in lignin biosynthesis, their regulatory mechanism remains to be elucidated.

Aphids are major agricultural pests that directly cause damage to plants by depriving them of nutrients, and aphids are also vectors for viral pathogens^17^. Chrysanthemum (*Chrysanthemum morifolium*) is a valuable ornamental species that is vulnerable to *Macrosiphoniella sanborni*. Aphid infestations hinder not only vegetative growth but also the quality and yield of chrysanthemum flowers. Our previous studies showed that chrysanthemum has evolved a number of strategies to cope with aphid infestation, such as increased defensive enzyme activity^18^ and gene expression reprogramming^19,20^. We previously found that *CmMYB19*, a chrysanthemum R2R3-MYB gene, improved the resistance of chrysanthemum to aphids by promoting the biosynthesis of lignin^21^. In the present study, we isolated chrysanthemum *CmMYB15*, a gene homologous to *AtMYB15*, and found that it played a role in lignin biosynthesis and aphid resistance by regulating several downstream genes different from *CmMYB19*. This study will lay a foundation for the understanding of the physical defense mechanisms of chrysanthemum when challenged by aphids.

## Results

### Isolation and sequence analyses of CmMYB15

**Fig. 1:**
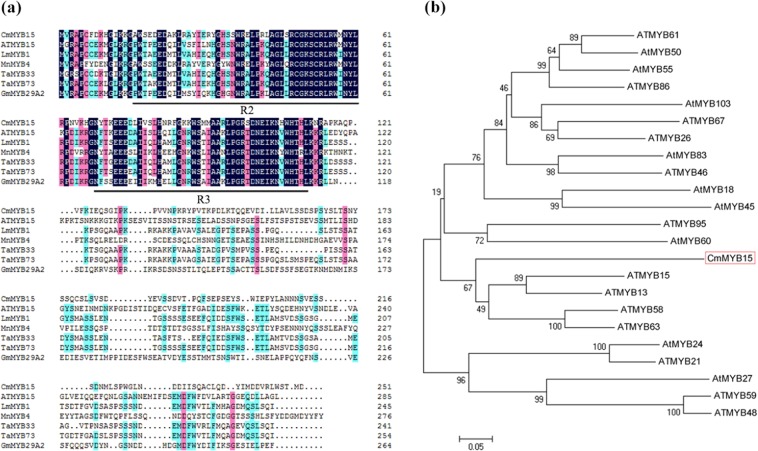
Putative peptide sequences of CmMYB15 and other MYB proteins. **a** Alignment of the putative amino acid sequence of CmMYB15 (boxed) with that of related MYB proteins that feature an R2MYB domain or an R3MYB domain (underlined), **b** Phylogenetic analysis of the relationships between CmMYB15 and other MYB proteins from Arabidopsis. The sequence details are as follows: AtMYB15 (AT3G23250.1), LmMYB1 (ADN96004.1), MnMYB4 (XP_010095860.1), TaMYB33 (AEO21928.1), TaMYB73 (XP_020169006.1), GmMYB29A2 (BAA81732.1), ATMYB61 (AT1G09540.1), AtMYB50 (AT1G57560.1), AtMYB55 (AT4G01680.1), ATMYB86 (AT5G26660.1), AtMYB103 (AT1G63910.1), ATMYB67 (AT3G12720.1), ATMYB26 (AT3G13890.1), AtMYB83 (AT3G08500.1), ATMYB46 (AT5G12870.1), AtMYB18 (AT4G25560.1), AtMYB45 (AT3G48920.1), ATMYB95 (AT1G74430.1), AtMYB60 (AT1G08810.1), ATMYB13 (AT1G06180.1), ATMYB58 (AT1G16490.1), ATMYB63 (AT1G79180.1), AtMYB24 (AT5G40350.1), ATMYB21 (AT3G27810.1), AtMYB27 (AT3G53200.1), ATMYB59 (AT5G59780.3), and ATMYB48 (AT3G46130.1)

*CmMYB15* was isolated from ‘Nannong Xunzhang’ chrysanthemum. The *CmMYB15* (KT763374) gene consists of 890 bp, with a 756 bp open reading frame (ORF) encoding a 251 amino acid polypeptide. *CmMYB15* belongs to the R2R3-MYB subfamily, as it has two conserved MYB domains: an R2MYB domain between aa 16–61 and an R3MYB domain between aa 69–112 (Fig. 1a). Phylogenetic analysis indicated that this gene has 32.73% similarity with AtMYB15 (Fig. 1b).

### CmMYB15 is localized to the cell nucleus

**Fig. 2:**
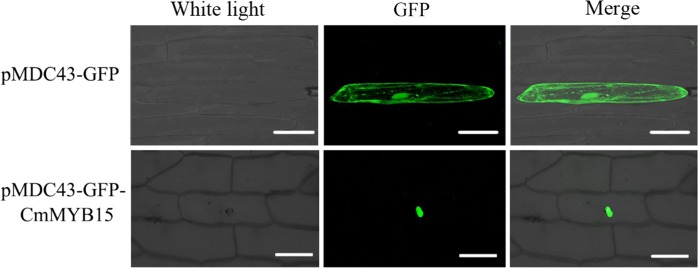
Subcellular localization of CmMYB15. Bar  = 100  μm

To determine the subcellular localization of CmMYB15, 2 × 35S::CmMYB15-GFP and a negative control, 2 × 35S::GFP, were separately transformed into onion epidermal cells. GFP signals were detected in the nucleus of the cells transformed with 2 × 35S::CmMYB15-GFP, indicating that CmMYB15 was nuclear localized. The GFP signal was detected in both the cytoplasm and nucleus of the cells transformed with 2 × 35S::GFP (positive control) (Fig. 2).

### Transcriptional activation of CmMYB15 and the binding ability of CmMYB15 with AC elements

**Fig. 3:**
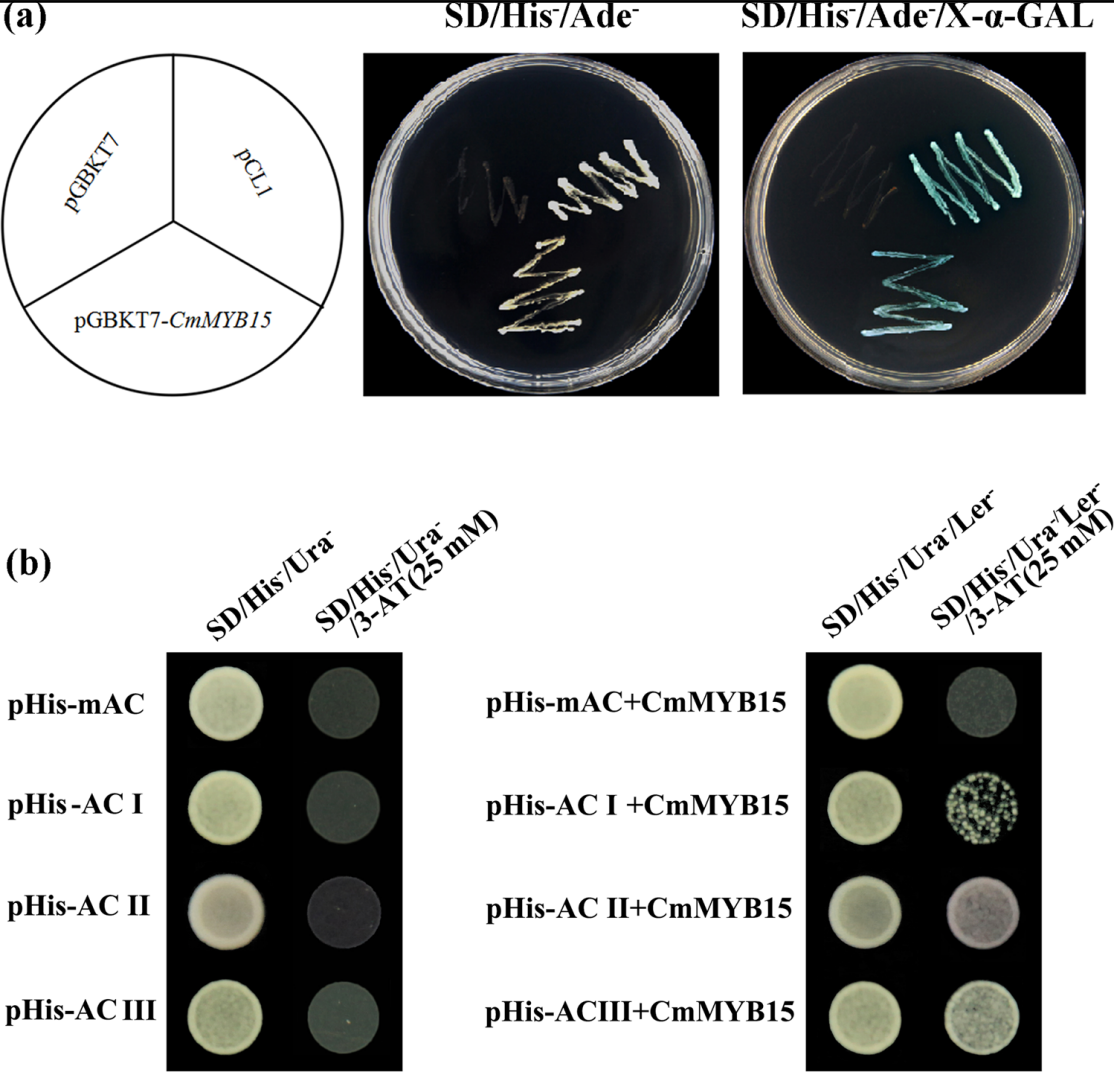
Transcriptional activation activity analyses of CmMYB15 and the DNA-binding assay of AC elements with CmMYB15. **a** The transcriptional activity analysis of CmMYB15 via a yeast assay system, in which pCL1 is a positive control and pGBKT7 is a negative control. **b** Yeast one-hybrid (Y1H) assay of CmMYB15 with the three kinds of AC elements: AC-I, AC-II, and AC-III, as well as their mutated form mAC

Yeast harboring pGBKT7-*CmMYB15*, or a pCL1 positive control, grew well on SD/His^-^/Ade^-^ medium and turned blue on the SD/His^-^/Ade^-^ medium with X-α-gal. The negative control transformed with pGBKT7 was unable to grow on the SD/His^-^/Ade^-^ medium (Fig. 3a), indicating that CmMYB15 has transcriptional activation activity. A yeast one-hybrid assay showed that cells transformed with pHISi-AC-I, pHISi-AC-II or pHISi-AC-III and its corresponding mutated form pHISi-mAC grew well on SD/His^-^/Ura^-^ medium but not on medium with 25 mM 3-amino-1,2,4-triazole (3-AT) (Fig. 3b). Bait Y1H cells (those harboring AC elements or a mAC element) transformed with pGADT7-CmMYB15 could grow on SD/His^-^/Ura^-^/Leu^-^ medium. In contrast to cells expressing pHISi-mAC, cells expressing pHISi-AC-I, pHISi-AC-II, or pHISi-AC-III could grow on medium supplemented with 25 mM 3-AT (Fig. 3b), suggesting that CmMYB15 could bind all three types of AC elements.

### The gene *CmMYB15* is highly expressed in leaves and stems and is induced by aphid infestation

**Fig. 4:**
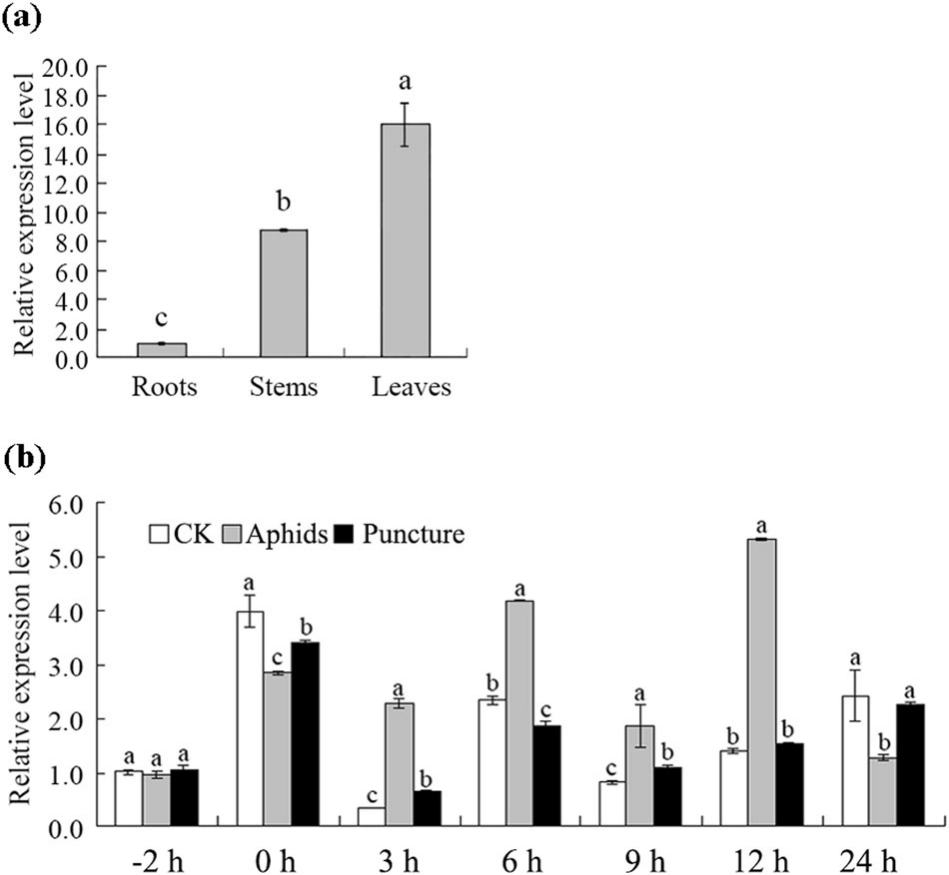
Expression of *CmMYB15* in different organs of chrysanthemum plants and under aphid and puncture treatment, as revealed by quantitative real-time PCR. **a** Expression of *CmMYB15* in the roots, stems and leaves of wild-type chrysanthemum, **b** Expression of *CmMYB15* in response to aphid treatment and puncture treatment in the third fully expanded leaf counted from the apex. The bars indicate the standard errors

The expression of *CmMYB15* was detected in the root, stem and leaf tissues of ‘Nannong Xunzhang’. *CmMYB15* had the lowest abundance in the roots, while it was highly expressed in the leaves and stems. The expression levels in the stems and leaves were 8.63 times and 15.79 times the level in the roots, respectively (Fig. 4a). In response to aphid infestation, the expression level of *CmMYB15* in the aphid-infested plants was 6.84 times greater than that in the control plants at 3 h. During 3 h to 12 h after aphid infestation, the expression level of *CmMYB15* remained significantly higher than that of the control. The expression level of *CmMYB15* was affected by mock puncture except at −2 h, 12, and 24 h; however, the changes in the expression level of *CmMYB15* in response to puncture were much less drastic than those in response to infestation by aphid (Fig. 4b).

### Overexpression of *CmMYB15* inhibited the population of aphids on chrysanthemum

**Fig. 5:**
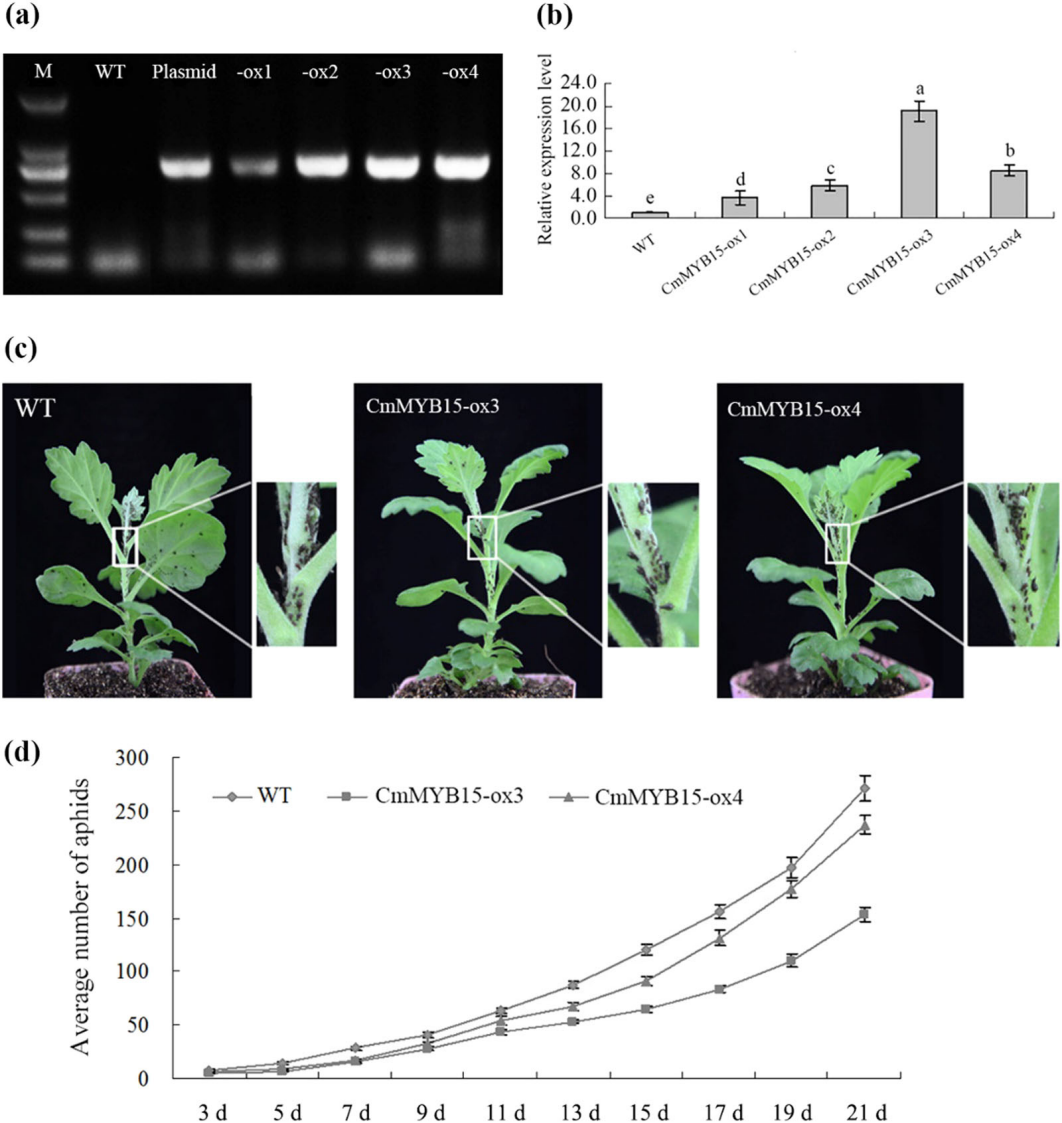
The aphid resistance of wild-type chrysanthemum and *CmMYB15-*overexpressing chrysanthemum. **a** Identification of *CmMYB15*-overexpressing chrysanthemum plants by PCR, in which the plasmid pMDC43-2 × 35 S::CmMYB15 acted as a positive control and wild-type (WT) acted as a negative control. -ox1, -ox2, -ox3 and -ox4 represent transgenic plants overexpressing *CmMYB15*, **b** transcription level of *CmMYB15* in wild-type and *CmMYB15-*overexpressing chrysanthemum plants, **c** differential proliferation of aphids between *CmMYB15-*overexpressing and nontransgenic plants, **d** average number of aphids on *CmMYB15-*overexpressing and wild-type plants measured at 3–21 days after aphid infestation

Putative *CmMYB15* overexpression lines were identified by PCR using *HptII* primers (Fig. 5a), and the transcriptional level of *CmMYB15* was measured by qRT-PCR using *CmMYB15*-specific primers (Fig. 5b). Two independent transgenic lines, CmMYB15-ox3 and CmMYB15-ox4, which presented a higher gene expression level of *CmMYB15*, were selected for additional aphid resistance assays. The aphids were more widely distributed on the WT plants than on transgenic lines at 21 days after infestation (Fig. 5c). At 11 days after infestation, the number of aphids began to increase rapidly in the control group (WT) plants and reached an average of 270.83 aphids per plant, while the number of aphids on the CmMYB15-ox3 and CmMYB15-ox4 plants were, on average, 153.45 and 236.80 per plant on day 21 (Fig. 5d). *CmMYB15*-overexpressing plants had a lower average multiplication rate (MR) of 30.69 for CmMYB15-ox3 and 47.36 for CmMYB15-ox4 in comparison to the WT MR of 54.17. Compared to the WT plants, the overexpression lines had higher average inhibition rates (IRs). The IRs for CmMYB15-ox3 and CmMYB15-ox4 in comparison to WT were 43.34% and 12.57%, respectively (Table 1).

**Table 1:** Comparison of aphid populations on wild-type plants and transgenic chrysanthemum plants overexpressing *CmMYB15* after infestation

Plants	Multiplication rate (MR) of the aphid population	Inhibition rate (IR) of the aphid population %
WT	54.17 ± 2.286^a^	0
CmMYB15-ox3	30.69 ± 1.422^c^	43.34
CmMYB15-ox4	47.36 ± 1.724^b^	12.57

Multiplication rate (MR) of the aphid population was calculated by *N*_*21*_*/5*, in which *N*_*21*_ represents the average number of aphids on WT plants or transgenic plants on the 21st day after aphid infestation. The inhibition rate (IR) of aphids on the transgenic plants was given by the formula (*N*_*W*_-*N*_*O*_)/*N*_*W*_ × 100, in which *N*_*W*_ and *N*_*O*_ represents the number of aphids on the WT and on the *CmMYB15*-overexpressing plants at 21 days after aphid infestation, respectively^22^

The different letters (^a^, ^b^, ^c^) represent significantly different aphid multiplications between the wild-type and *CmMYB15*-overexpressing plants

### Overexpression of *CmMYB15* enhanced the lignin content and expression of lignin biosynthesis genes

**Fig. 6:**
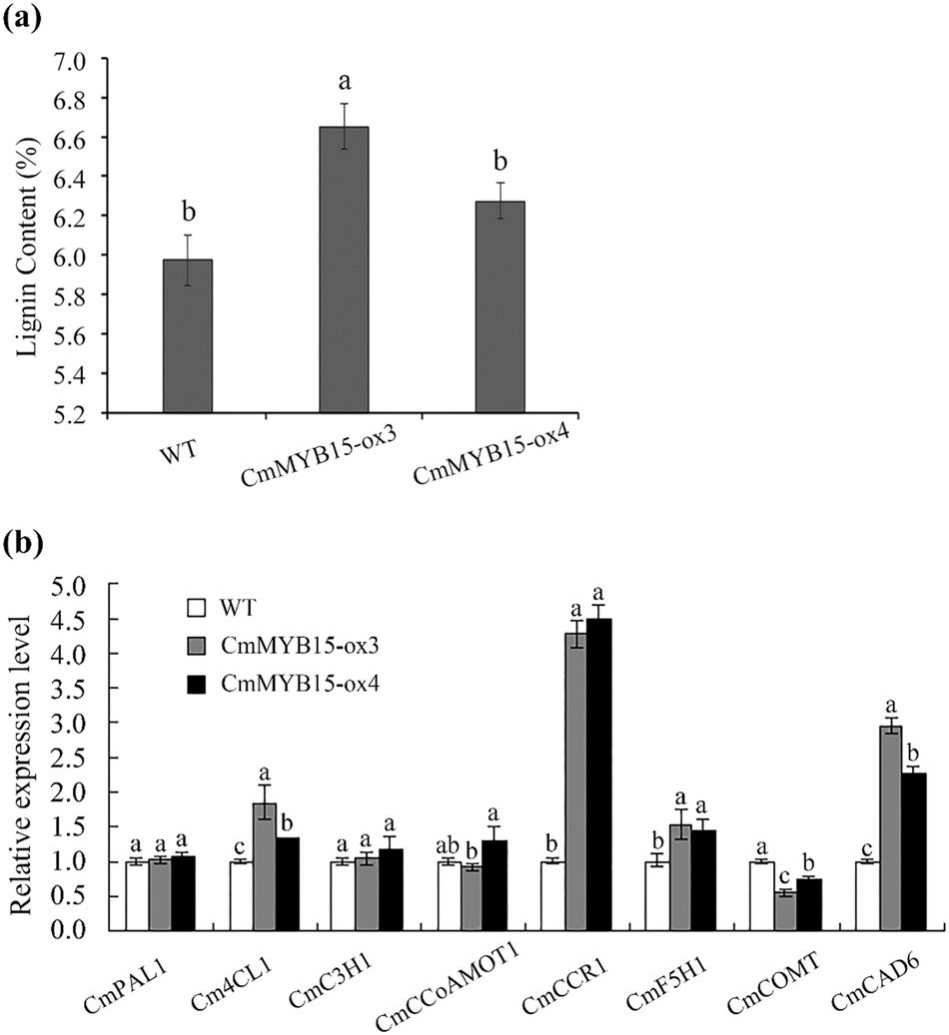
The lignin content and expression levels of lignin biosynthesis genes in *CmMYB15*-overexpressing plants. **a** The lignin content of wild-type and *CmMYB15*-overexpressing plants, **b** expression levels of eight lignin biosynthesis genes in wild-type and *CmMYB15*-overexpressing plants. The bars indicate the standard errors

The acetyl bromide procedure^23^ was used to analyze the lignin content of two *CmMYB15* overexpression lines. The lignin content of CmMYB15-ox3 and CmMYB15-ox4 was 6.56% and 6.27% respectively, while the lignin content of the WT was 5.97% (Fig. 6a).

The expression levels of genes in the lignin biosynthesis pathway, including *CmPAL1*, *Cm4CL1*, *CmC3H1*, *CmCSE*, *CmCCoAMOT1*, *CmCCR1*, *CmF5H1*, *CmCOMT*, and *CmCAD6*, were also quantified. The expression levels of *Cm4CL1*, *CmCCR1*, *CmF5H1*, and *CmCAD6* were higher in the overexpression plants than in the WT plants; among these, compared to the other genes analyzed, the expression of *CmCCR1* and *CmCAD6* increased significantly (Fig. 6b).

## Discussion

### *CmMYB15* is a MYB transcription factor

MYB transcription factors, which are widely distributed in plants, play an important role in regulating a variety of processes in plants^1^. MYBs act as transcription activators or repressors to regulate the transcription of downstream genes. Of all the divisions of plant MYB proteins, including 4R-MYB, 3R-MYB, R2R3-MYB and MYB-related types, R2R3-MYB proteins account for the majority^24^. Most R2R3-MYB and 3R-MYB subfamilies in Arabidopsis are divided into 16 groups with different functions, such as involvement in stress responses, primary and secondary metabolism, lignin biosynthesis or wall deposition, cell wall thickening, cell differentiation or embryogenesis, the cell cycle, anther development, stomatal development and meristem formation^3,25^. Previously, only a few MYB transcription factors in chrysanthemum had been isolated and analyzed. Among them, chrysanthemum *CmMYB1* is a repressor that reduces the lignin content and represses flavonoid synthesis in Arabidopsis^26^. Chrysanthemum *CmMYB2* enhances the salinity and drought stress of Arabidopsis; additionally, the ABA sensitivity and flowering period of plants is affected by this MYB^27^. Chrysanthemum *CmMYB6* was found to be involved in anthocyanin biosynthesis^28^. In this study, a new MYB family gene in chrysanthemum, *CmMYB15*, was isolated. Sequence analysis showed that *CmMYB15* contains two conserved MYB domains, which are typical of R2R3-MYB genes. Cluster analysis showed that *CmMYB15* is closely related to *AtMYB15*, *AtMYB13*, *AtMYB58*, and *AtMYB63*. These genes are thought to belong to a group related to stress responses^29^. In addition, CmMYB15 was found by GFP localization analysis to be expressed in the cell nucleus, which is expected given its role in transcriptional activation.

### Overexpression of *CmMYB15* enhanced aphid resistance in chrysanthemum

Plants are commonly exposed to many forms of abiotic and biotic stresses during growth. In response to these stresses, plants have evolved various defense mechanisms. MYBs are involved in the response to abiotic stress, including salt, drought, and high or low temperature^30–32^. For example, *AtMYB20* can enhance salt tolerance^33^, while *AtMYB73* suppresses salt overly sensitive (SOS) genes and is a negative regulator of salt responses^34^. Several MYBs, such as *AtMYB74* and *AtMYB88*, participate in both salt and drought stress responses^35–37^. *AtMYB15*, a homologous gene of *CmMYB15*, enhances drought and salt tolerance by improving sensitivity to abscisic acid, but it weakened the freezing tolerance of Arabidopsis by suppressing the expression of *CBF1/DREB1*, which activated cold-responsive genes^38,39^. On the other hand, the *Fagopyrum tataricum* MYB gene *FtMYB15* enhances the synthesis of anthocyanins and proanthocyanidins, which help resist abiotic stress^40^. Similarly, *SlMYB75* in tomato also contributes to the accumulation of anthocyanins and participates in several stresses responses^41^.

At present, the MYBs involved in the response to biotic stresses such as pathogen attack and pest feeding have received increased amounts of attention. By promoting the biosynthesis of salicylic acid, *AtMYB96* induces pathogen resistance in Arabidopsis^42^. The role of MYBs in insect defense has been recognized; for example, *AtMYB102* is expressed locally at the feeding sites of *Pieris rapae* in damaged leaves, which then activates the expression of defense-related genes and cell wall modification genes^43^. Overexpression of *AtMYB12* in *Nicotiana tabacum* enhances the expression of phenylpropanoid pathway genes and the accumulation of flavonols, especially rutin, consequently leading to insect resistance^31^. In the present study, *CmMYB15* was significantly induced by aphid infestation. *CmMYB15*-overexpressing plants exhibited a lower average multiplication rate and higher average inhibition ratio of aphids, in which CmMYB15-ox3 lines, which presented a relatively high expression of the *CmMYB15* gene, showed a higher IR than CmMYB15-ox4 lines, which presented relatively low *CmMYB15* expression. These results indicated that *CmMYB15* contributed to the resistance of chrysanthemum to aphids.

### *CmMYB15* enhanced aphid resistance by increasing lignin synthesis

When faced with phytophagous pests, plants can enhance their physical structures and alter their secondary metabolites to hinder the invasion of pests or inhibit pest growth and reproduction. Research in rice (*Oryza sativa*) has demonstrated that three MYB genes, *MYB30*, *MYB55*, and *MYB110*, which are induced by pathogen infestation, are involved in plant primary immunity (known as a microbe-associated molecular pattern) by specifically inducing monolignol pathway genes^44^. In Arabidopsis, recent research has demonstrated that *AtMYB102* promotes ethylene (ET) biosynthesis by upregulating ET biosynthesis pathway genes and increases the susceptibility of plants to aphids^45^. Phloem-based defense (PBD) regulated by ethylene (ET) and MYB transcription factors is a mechanism that has been studied substantially in plants with specific regard to how plants are resistant to insects such as aphids. Sieve-specific phloem proteins (PP) can plug sieve pores and prevent aphids feeding on the phloem, which is a physical plant defense mechanism^46^. In wheat (*Triticum aestivum*), *TaMYB19*, *TaMYB29*, and *TaMYB44* act as coregulators of PBD, which is activated by the infestation of English grain aphids via crosstalk with the ethylene signaling pathway^46^. Three Arabidopsis mutants, *atmyb15*, *atmyb38*, and *atmyb44*, are susceptible to aphid infestation due to the disruption of ethylene signaling^47^, with *AtMYB44* playing a dominant role in activating EIN2-affected defense, an ethylene signaling regulation defense mechanism, to control resistance to the green peach aphid *Myzus persicae* Sulzer. This defense requires ethylene-induced resistance involving the PBD response^48–50^. However, the mechanism of *AtMYB15* in aphid resistance has not been further studied. Recently, it was determined that *AtMYB15* regulates the biosynthesis of G-lignin to promote defense-induced lignification in Arabidopsis in response to pathogens, with *AtMYB15* interfering with flg22, the bioactive epitope of bacterial flagellin^6^. Our previous study showed that *CmMYB19*, an aphid stress response gene, promoted lignin synthesis and resistance to aphids^21^. Here, we found that *CmMYB15*, a member of the MYB family, also enhanced aphid resistance and lignin synthesis. However, different sets of lignin genes were induced downstream in *CmMYB19-* and *CmMYB15*-overexpressing plants. *Cm4CL1* and *CmCCR1* were induced in both *CmMYB19* and *CmMYB15* overexpression plants, while *CmF5H1* and *CmCAD6* were induced only in the *CmMYB15* overexpression plants. *CmPAL1*, *CmC4H*, *CmHCT* and *CmCCoAOMT1* were enhanced by overexpression of *CmMYB19*^21^; however, the expression level of *CmMYB19* was relatively little affected by *CmMYB15* (Fig. S1), suggesting that the regulatory mechanisms of lignin biosynthesis of *CmMYB15* and *CmMYB19* were different from each other to some extent. We suppose that *CmMYB15* might mainly regulate the synthesis of lignin monomers and that *CmMYB19* might contribute to the very upstream steps of lignin biosynthesis and the assembly of lignin monomers; this speculation should be validated in our ongoing research.

Yeast assays showed that CmMYB15 was able to bind to the AC elements in promoter regions of some lignin pathway genes, suggesting a role for CmMYB15 in the activation of lignin pathway genes. However, whether the elevation of genes involved in lignin biosynthesis is directly or indirectly regulated by *CmMYB15* is not currently known. In conclusion, increasing *CmMYB15* expression could lead to changes in lignin content, thereby enhancing the aphid resistance of chrysanthemum, which provides much promise for improving physical defense to aphids.

## Materials and methods

### Plant materials

‘Nannong Xunzhang’ (aphid resistant) and ‘Jinba’ (aphid susceptible) chrysanthemum plants were obtained from the Chrysanthemum Germplasm Resource Preserving Centre (Nanjing Agricultural University, China). The plants were grown under conditions of 25 °C and 100 μmol m^−2^ s^−1^, with a 16/8 h light/dark photoperiod.

### Gene isolation and sequence analysis

Total RNA from ‘Nannong Xunzhang’ chrysanthemum was isolated using RNAiso Plus Reagent (TaKaRa, Japan). First-strand cDNA was synthesized by M-MLV reverse transcriptase (TaKaRa, Japan). The cDNA fragments of *CmMYB15* were amplified using Phusion High-Fidelity DNA Polymerase (ThermoFisher, USA) with gene-specific primers (Supplementary Table S1) based on the transcriptome data of ‘Nannong Xunzhang’^20^. 3′-RACE (3′-random amplification of cDNA ends) and 5′-RACE were then performed to obtain the full-length cDNA as described previously^21^. The ORF of *CmMYB15* was amplified using the gene-specific primers CmMYB15-F/R (Supplementary Table S1).

The homologous sequences of *CmMYB15* were retrieved and analyzed using online BLAST tools from the NCBI (National Center for Biotechnology Information, USA). The sequences of MYB proteins were aligned using DNAMAN and Muscle software. A phylogenetic tree of CmMYB15 with its homologs in Arabidopsis was constructed using MEGA 7.0 with the maximum likelihood method (bootstraps = 1000).

### Subcellular localization of CmMYB15

The ORF of *CmMYB15* was ligated into the vector pENTR1A using *Kpn* I and *Xho* I restriction enzymes to generate a pENTR1A-*CmMYB15* vector. A pMDC43-*CmMYB15* vector driven by the 2 × 35S promoter was then constructed via recombination of pENTR1A-*CmMYB15* with pMDC43-GFP using LR Clonase II enzyme mix (Invitrogen, USA). By particle bombardment (PDS-1000; Bio-Rad, USA), the plasmid DNA of pMDC43-CmMYB15 was transformed into onion epidermal cells^51^. The GFP signal of the transformed cells was detected by laser scanning confocal microscopy (LSCM, Leica, Germany) after an overnight dark incubation at 22 °C on Murashige and Skoog (MS) medium.

### Yeast one-hybrid assay for the transcriptional activation activity and binding of AC cis-elements by CmMYB15

To detect the transcriptional activity of CmMYB15, pGBKT7-*CmMYB15* was constructed via recombination using pENTR1A-*CmMYB15* as the donor vector and pGBKT7 as the destination vector with the aid of LR Clonase II enzyme mix (Invitrogen, USA). pGBKT7-*CmMYB15*, pGBKT7 (an empty vector, as the negative control) and a positive control (pCL1) were transformed separately into Y2H competent cells (Clontech, USA) containing the GAL4/UAS system. The transformed cells were cultured as described previously^21^.

To identify whether CmMYB15 could bind AC elements, a yeast one-hybrid assay was performed using the Matchmaker Gold Yeast One-Hybrid Library Screening System (Clontech, USA) according to the manufacturer’s protocol. Three forms of AC elements, i.e., AC-I (ACCTACC), AC-II (ACCAACC) and AC-III (ACCTAAC), are available thus far^52^. Therefore, the fragments containing three tandem repeat AC elements, AC-I (5′-TCCACCTACCTCCACCTACCTCCACCTACCCC-3′), AC-II (5′-TCCACCAACCCCTCCACCAACCCCTCCACCAACCCC-3′), and AC-III (5′-TCCACCTAACTCCACCTAACTCCACCTAACCC-3′), respectively, and their mutated form, mAC (5′-TCCAAATATTTCCAAATATTTCCAAATATTCC-3′), were assembled into a pHISi vector at the *EcoR* I and *Xba* I restriction enzyme cutting sites. The plasmids were transformed into a Y1H Gold line after linearization by an *Apa* I restriction enzyme. The binding assay was performed as described previously^21^.

### Gene expression analysis by quantitative real-time PCR (qRT-PCR)

To analyze the expression profiles of *CmMYB15* in different tissues, the roots, the fourth internode of the stem and the third fully expanded leaf counted from the apex were harvested from ‘Nannong Xunzhang’.

To dissect the expression profiles of *CmMYB15* in response to aphid infestation, aphid infestation was performed as described by Xia et al.^20^. The time point when aphids were infested was defined as −2 h; thus, 0 h refers to 2 h after infestation. To mimic piercing by an aphid, a puncture treatment was performed on the third leaf of another group of plants as described previously^51^. A group of untreated plants served as the control (CK). All the treated leaves were covered by transparent and ventilated petri dishes^19^. For RNA isolation, leaves from each treatment were harvested from three individual plants at −2, 0, 3, 6, 9, 12, and 24 h. The aphids were removed when the leaves were harvested. Each test included three biological replicates.

qRT-PCR was performed using SYBR Premix Ex Taq II (TaKaRa, Japan) according to the manufacturer’s instructions. The gene *Elongation Factor 1α* (*CmEF1α*, KF305681) was used as the reference gene^53^. The qRT-PCR data were analyzed using the 2^− ΔΔCT^ method^54^.

### Overexpression of *CmMYB15* in ‘Jinba’ chrysanthemum

To identify the function of *CmMYB15*, pMDC43-2 × 35S::CmMYB15 was transformed into *Agrobacterium tumefaciens* strain EHA105 using the freeze-thaw method. Information on the in vitro regeneration of ‘Nannong Xunzhang’ chrysanthemum is not available; thus, a *CmMYB15-*overexpressing ‘Jinba’ chrysanthemum was produced by Agrobacterium-mediated transformation as described previously^55^. Hygromycin-resistant shoots were rooted on MS medium containing hygromycin B, and the putative transgenic plants were identified by PCR using *HptII* primer pairs (Supplementary Table S1). The expression levels of *CmMYB15* in the putative overexpression lines and wild-type (WT) plants were measured using the primer pair CmMYB15-qF/qR (Supplementary Table S1).

### The resistance of *CmMYB15-*overexpressing chrysanthemum plants to aphid

Two independent overexpression lines were used for an aphid resistance assay according to the methods of Hu et al.^56^. Transgenic plants and WT ‘Jinba’ plants at the 6–8-leaf stage were used for aphid resistance assays. The infestation of aphids was performed according to the method of Li et al.^55^. Each infestation assay contained ten plants with three biological replications. The number of aphids was counted every 2 days. The average multiplication rate (MR) and average IR of the aphid population were calculated based on the aphid data counted over 21 days^21^.

### The determination of lignin content and expression levels of lignin biosynthesis genes in *CmMYB15-*overexpressing plants

The fourth internode of the overexpressing lines and WT ‘Jinba’ plants at the 14-to-16-leaf stage was sampled, dried and ground. The lignin content was analyzed using the acetyl bromide procedure. The samples were prepared following the description by Foster et al.^23^ The absorbance at 280 nm was determined using a microplate reader. The lignin content was then determined using the formula %ABSL = [abs × (2 ml × 100%)]/[(15.69 × 0.539 cm) × weight], where abs (absorbance) represents the average absorbance of 3 reads.

The fourth internode of the overexpressing lines and ‘Jinba’ plants at the 14-to-16-leaf stage was harvested. The expression of lignin biosynthesis genes, including *CmPAL1*, *Cm4CL1*, *CmC3H1*, *CmCSE*, *CmCCoAMOT1*, *CmCCR1*, *CmF5H1*, *CmCOMT*, and *CmCAD6*, was measured by qRT-PCR as mentioned above using the primers listed in Supplementary Table S1.

### Statistical analysis

Tukey’s multiple range test (*P* < 0.05) and one-way analysis of variance were performed to determine the significance of the data. SPSS v19.0 was used for all statistical analyses.

## Supplementary information

Supplementary information.
