# Supplementary material for: Overexpression of CmMYB15 provides chrysanthemum resistance to aphids by regulating the biosynthesis of lignin
Source: Hortic Res. 2019 Jul 11;6:84. doi: 10.1038/s41438-019-0166-y (PMC6804602; doi:10.1038/s41438-019-0166-y)
Supplement: Supplementary file 1 — Supplementary information. [file 41438_2019_166_MOESM1_ESM.docx]

**Supplemental table 1. Primer names and sequences**

| Primer name | Sequence (5′ to 3′) |
| --- | --- |
| CmMYB15-F | ATGGTTAGGGCACCTTGTTTCG |
| CmMYB15-R | ATCCATAGTTGACCATAAACGCACA |
| CmMYB15-qF | CAAGAGGTTGACATTTTATTGGCAG |
| CmMYB15-qR | TCACTTGATGATTCCACGCTATTGT |
| CmPAL1-qF | CCCCAACAGGATCAAGGCAT |
| CmPAL1-qR | TTGTCGAACTCTTCACCCGG |
| Cm4CL1-qF | TTCATCGTTGACCGGCTCAA |
| Cm4CL1-qR | TCCTCCGTCAAACTTGAGCC |
| CmC3H1-qF | CGCCAATGTCAAGGTTGGTG |
| CmC3H1-qR | GTGGCAGGATCTCGAGCTAC |
| CmCCoAOMT-qF | TGGCTGCCGATCCAAGAATT |
| CmCCoAOMT-qR | GACTCGACGGCAAAGGGTAA |
| CmCCR1-qF | CATTTGTGTCACCGGTGCTG |
| CmCCR1-qR | AACGGTTCCTCGAACAGCAT |
| CmF5H1-qF | CTTCATTGACCCCGCTGGAT |
| CmF5H1-qR | TTTCGGCATCTTCATCGCCT |
| CmCOMT-qF | TGTCATGACTGGAGTGACGC |
| CmCOMT-qR | GGAAGAATGCATTCCGCGAC |
| CmCAD6-qF | CCGATGGAATGTCTCCCGAG |
| CmCAD6-qR | ACGTGATGTCCCATCGCTTT |
| CmEF1α-F | TTTTGGTATCTGGTCCTGGAG |
| CmEF1α-R | CCATTCAAGCGACAGACTCA |
| HptII-F  -F | CTTCTACACAGCCATCGGTCCAG |
| HptII-R  -R | CGGAAGTGCTTGACATTGGGGAG |


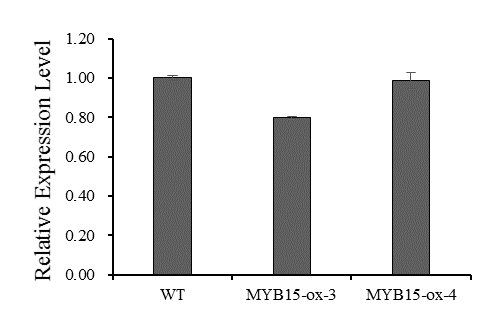


Fig. S1 The expression of *CmMYB19* in *CmMYB15*-o
